# Chondroitinase ABC Administration Facilitates Serotonergic Innervation of Motoneurons in Rats With Complete Spinal Cord Transection

**DOI:** 10.3389/fnint.2022.881632

**Published:** 2022-06-30

**Authors:** Masahito Takiguchi, Kanae Miyashita, Kohei Yamazaki, Kengo Funakoshi

**Affiliations:** ^1^Department of Neuroanatomy, Yokohama City University School of Medicine, Yokohama, Japan; ^2^Yokohama City University School of Medicine, Yokohama, Japan

**Keywords:** complete transection, perineuronal net, chondroitin sulfate proteoglycan, spinal cord injury, serotonin

## Abstract

Chondroitinase ABC (ChABC) is an enzyme that degrades glycosaminoglycan side-chains of chondroitin sulfate (CS-GAG) from the chondroitin sulfate proteoglycan (CSPG) core protein. Previous studies demonstrated that the administration of ChABC after spinal cord injury promotes nerve regeneration by removing CS-GAGs from the lesion site and promotes the plasticity of spinal neurons by removing CS-GAGs from the perineuronal nets (PNNs). These effects of ChABC might enhance the regeneration and sprouting of descending axons, leading to the recovery of motor function. Anatomical evidence, indicating that the regenerated axons innervate spinal motoneurons caudal to the lesion site, however, has been lacking. In the present study, we investigated whether descending axons pass through the lesion site and innervate the lumbar motoneurons after ChABC administration in rats with complete spinal cord transection (CST) at the thoracic level. At 3 weeks after CST, 5-hydroxytryptamine (5-HT) fibers were observed to enter the lesion in ChABC-treated rats, but not saline-treated rats. In addition, 92% of motoneurons in the ventral horn of the fifth lumbar segment (L5) in saline-treated rats, and 38% of those in ChABC-treated rats were surrounded by chondroitin sulfate-A (CS-A) positive structures. At 8 weeks after CST, many 5-HT fibers were observed in the ventral horn of the L5, where they terminated in the motoneurons in ChABC-treated rats, but not in saline-treated rats. In total, 54% of motoneurons in the L5 ventral horn in saline-treated rats and 39% of those in ChABC-treated rats were surrounded by CS-A-positive structures. ChABC-treated rats had a Basso, Beattie, and Bresnahan (BBB) motor score of 3.8 at 2 weeks, 7.1 at 3 weeks, and 10.3 at 8 weeks after CST. These observations suggest that ChABC administration to the lesion site immediately after CST may promote the regeneration of descending 5-HT axons through the lesion site and their termination on motoneurons at the level of caudal to the lesion site. ChABC administration might facilitate reinnervation by degrading CS-GAGs around motoneurons. Motor function of the lower limbs was significantly improved in ChABC-treated rats even before the 5-HT axons terminated on the motoneurons, suggesting that other mechanisms may also contribute to the motor function recovery.

## Introduction

Traumatic brain injury and spinal cord injury (SCI) lead to the formation of scar tissue at the lesion site in the central nervous system. The scar that develops after stab injury in mammals comprises fibrous tissue in the lesion core and glial tissue in the surrounding parenchyma (Klapka and Müller, 2006; Kawano et al., 2012). Glial tissue represents severe reactive astrogliosis, which creates a chemical barrier containing various extracellular matrix (ECM) molecules that entrap axons and limit their ability to regrow over long distances. Among these ECM molecules, chondroitin sulfate proteoglycans (CSPGs) are considered major growth-inhibitory factors (Snow et al., 1990; McKeon et al., 1991, 1995; Silver and Miller, 2004). CSPGs are a subset of proteoglycans made up of core proteins and glycosaminoglycan side-chains of chondroitin sulfate (CS-GAG) that covalently bind to core proteins. The CS-GAGs are largely responsible for inhibiting axonal regeneration (Dou and Levine, 1995; Bradbury et al., 2002; Laabs et al., 2007). CS-GAGs directly interact with receptors expressed on the surface of injured axons to activate signal pathways for growth inhibition. These receptors include protein tyrosine phosphatases, such as protein tyrosine phosphatase σ and leukocyte common antigen-related phosphatase, and Nogo receptors (Miller and Hsieh-Wilson, 2015).

Chondroitinase ABC (ChABC) is a bacterial enzyme that degrades CS-GAGs from the CSPG core protein. Previous studies revealed that the administration of ChABC after SCI removed CS-GAGs at the lesion site and promoted nerve regeneration in long tracts entering the lesion site (Bradbury et al., 2002; Yick et al., 2004; Barritt et al., 2006; Huang et al., 2006; García-Alías et al., 2008; Bradbury and Carter, 2011). ChABC administration restores motor function provided by the spinal cord at levels below the lesion (Bradbury et al., 2002; Caggiano et al., 2005; Barritt et al., 2006; Huang et al., 2006; García-Alías et al., 2008; Tester and Howland, 2008; Bradbury and Carter, 2011). Many pre-clinical studies have demonstrated the efficiency of ChABC as a treatment for SCI (Muir et al., 2019). The mechanisms of functional recovery, however, are not known. The regeneration of descending tracts might not be involved in functional recovery. In cases of partial injury, hemisection, or contusion of the spinal cord, spared axons of descending tracts may maintain their projections to the caudal motor areas and contribute to the recovery of motor functions. Furthermore, the administration of ChABC degrades CSPGs in the perineuronal nets (PNNs) to promote the plasticity of spinal neurons and the sprouting of spared long tract axons (Massey et al., 2006; Tom et al., 2009; Starkey et al., 2012). Therefore, complete spinal cord transection (CST) is the only model that allows us to evaluate whether regenerating axons passing through the lesion site are involved in the recovery of motor function. Previous studies have revealed that ChABC administered after CST promotes axonal regeneration across the lesion site and significantly improves functional recovery (Huang et al., 2006; Cheng et al., 2015). How the motor function recovered, however, remains unclear because the projections of the regenerated axons to spinal neurons in the motor area were not evaluated.

In the present study, therefore, we investigated whether ChABC administration would promote the passage of regenerating axons through the lesion site and their termination on motoneurons at the lumbar level in rats with CST at the midthoracic level. We evaluated the regeneration of serotonergic fibers by immunohistochemistry using a 5-hydroxytryptamine (5-HT) antibody and the synaptic contacts of the regenerated fibers on motoneurons by immunohistochemistry using a synapsin I antibody. In addition, we investigated the effects of ChABC to degrade CSPGs in PNNs surrounding the motoneurons in the ventral horn at the lumbar level and the relationship between PNNs around motoneurons and the terminals of 5-HT nerves.

Glycosaminoglycan side-chains of chondroitin sulfate, a major inhibitory factor in CSPGs, are composed of repeating chondroitin sulfate (CS) disaccharide units formed by N-acetyl galactosamine (GalNAc) and glucuronic acid (GlcA) and modified by regional sulfation. CS disaccharides monosulfated in the four positions of the GalNAc residue are referred to as CS-A. Previous studies suggest that CS-A has negative effects on axonal outgrowth in rodent neurons (Dou and Levine, 1995; Wang et al., 2008). Therefore, we evaluated the effect of ChABC administration on CS-A degradation in PNNs.

## Materials and Methods

### Materials

Both male and female Wistar rats (*n* = 50, Japan SLC, Hamamatsu, Japan) were used in the present study. All experimental procedures were performed according to the standards established by the NIH Health Guide for the Care and Use of Laboratory Animals and the Policies on the Use of Animals and Humans in Research. The protocols were approved by the Institutional Animal Care and Use Committee of the Animal Research Center of Yokohama City University Graduate School of Medicine.

### Complete Spinal Cord Transection

The spinal cords of rats (*n* = 50) were transected at the 10th thoracic level (T10). Briefly, under isoflurane gas (1.5–2.0%) anesthesia, the spinal cord was completely transected with a sterile stainless surgical blade (No. 11) following partial laminectomy (T9-T10). After confirming that the spinal transection was complete, bleeding was stopped with a piece of gelatin sponge (Spongel; Astellas Seiyaku Co., Ltd., Tokyo, Japan). For rats with CST, a piece of gelatin sponge soaked in 50 μl of ChABC solution (1 U/ml in 50 mM in saline, Millipore Sigma, St. Louis, MO, United States) was placed at the lesion site (CST-ChABC rats: *n* = 25). As a control, a piece of gelatin sponge soaked in saline solution instead of ChABC was applied to the lesion site (CST-saline rats: *n* = 25). Gelatin sponges are water-insoluble and completely absorbed within 4–6 weeks when placed in soft tissues. In animal experiments, gelatin sponges are useful as a scaffold for the slow-release administration of chemicals (Okamoto et al., 2004; Takemoto et al., 2008). The muscles and fascia layers of the skin were closed with 6–0 nylon sutures. All rats with CST (CST rats) were housed individually in polycarbonate cages in a room maintained at 25 ± 1°C, with a 05:00 on/19:00 off light cycle. Severe dysuria due to the CST was addressed by the researcher by manually pressing on the bladder two times a day. A total of 16 CST rats did not survive 2 weeks after the CST procedure and were therefore not included in the analysis.

### Tissue Preparation for Immunohistochemistry

Complete spinal cord transection rats (*n* = 34) were deeply anesthetized with isoflurane at 2 weeks after CST (CST-ChABC: *n* = 6 and CST-saline: *n* = 6), 3 weeks after CST (CST-ChABC: *n* = 7 and CST-saline: *n* = 7), and 8 weeks after CST (CST-ChABC: *n* = 4 and CST-saline: *n* = 4), and transcardially perfused with normal saline followed by 4% paraformaldehyde in the 0.1 M phosphate buffer.

The spinal cords were dissected and postfixed with 4% paraformaldehyde overnight at 4^°^C. The tissues were then cryoprotected in 25% sucrose for 2 days and embedded in the optimal cutting temperature (OCT) compound *via* 2-methylbutane (isopentane) in liquid N_2_, and stored at –80°C until sectioning. Horizontal sections were cut at 20 μm with a cryostat (CM3050 S, Leica, Nussloch, Germany), attached to slides, and stored at –20°C.

### Immunohistochemistry

A series of all the sections of CST rats were used for immunohistochemistry. The slides were dried for 1 h and washed three times for 5 min each in 0.01 M phosphate-buffered saline (PBS). The sections were then incubated in PBS including 0.5% Tween 20 (PBST) for 30 min.

Chondroitin sulfate-A was detected using the monoclonal antibody clone 2H6, which recognizes multiple sequences containing CS-A units in CS chains (Deepa et al., 2007) and binds effectively in immunohistochemistry (Shimazaki et al., 2005). To label motoneurons, antibodies against choline acetyltransferase (ChAT) or NeuN that recognize α-motoneurons (Friese et al., 2009; Godinho et al., 2020) were used. After the blocking procedure with Block Ace (5%, UK-B80, DS Pharma Biomedical Co., Ltd., Suita, Japan), the sections were incubated in a moist chamber overnight at 4°C with primary antibodies as follows: (1) a mixture of goat polyclonal antibody against ChAT (1:100, AB144P, Merck KGaA, Darmstadt, Germany) and mouse monoclonal IgM antibody against CS-A (10 μg/ml, Clone 2H6, NU-07-001, Cosmo Bio Co., Ltd., Tokyo, Japan); (2) a mixture of mouse monoclonal IgG antibody against NeuN (1:200, MAB377, Merck KGaA), mouse monoclonal IgM antibody against CS-A (10 μg/ml, Clone 2H6, NU-07-001, Cosmo Bio), and goat polyclonal antibody against 5-HT (1:2,000, PA1-18017, Invitrogen, Carlsbad, CA, United States); (3) a mixture of goat polyclonal antibody against 5-HT (1:2,000, PA1-18017, Invitrogen) and mouse monoclonal IgG antibody against GAP43 (1:100, ab129990, Abcam, Cambridge, United Kingdom); (4) a mixture of goat polyclonal antibody against ChAT (1:100, AB144P, Merck KGaA), mouse monoclonal antibody against synapsin I (1:200, VAM-SV009, Stressgen Biotechnologies Corp., San Diego, CA, United States), and rabbit polyclonal antibody against 5-HT (1:200, S-5545, Sigma-Aldrich, Merck KGaA); and (5) a mixture of rabbit polyclonal antibody against collagen IV (1:200, ab6586, Abcam), and mouse monoclonal IgM antibody against CS-A (10 μg/ml, Clone 2H6, NU-07-001, Cosmo Bio), diluted with 1% normal donkey serum, 0.2% bovine serum albumin, and 0.1% NaN_3_ in 0.1 M PBST. After rinsing the sections several times with 0.01 M PBST, they were incubated for 2 h at room temperature with a mixture of secondary antibodies, i.e., cyanine Cy3-conjugated donkey anti-goat IgG (1:200, Jackson ImmunoResearch Laboratories, West Grove, PA, United States), Alexa Fluor 488-conjugated donkey anti-mouse IgG (1:200, Jackson ImmunoResearch Laboratories), Alexa Fluor 488-conjugated donkey anti-mouse IgM (1:200, Jackson ImmunoResearch Laboratories), and Alexa Fluor 647-conjugated donkey anti-mouse IgG (1:200, Jackson ImmunoResearch Laboratories).

The PNNs were also visualized by staining for the plant lectin *Wisteria Floribunda agglutinin* (*WFA*), a pan marker for CSPGs (Miyata et al., 2012; Irvine and Kwok, 2018; Sigal et al., 2019). For WFA staining, sections were incubated in fluorescein *WFA* lectin for 2 h (diluted 1:100 in PBS, FL-1351, Vector Laboratories, Inc., Burlingame, CA, United States).

The slides were then rinsed in PBS, and coverslips were mounted with a slow-fade reagent (SlowFade Gold antifade reagent, S36936, Invitrogen). Antibody specificity was verified by incubation with 0.5% normal mouse serum (Jackson ImmunoResearch Laboratories) or 0.5% normal goat serum (Jackson ImmunoResearch Laboratories) instead of the primary antibodies.

### Image Acquisition and Analysis

All the sections were digitally photographed using a Keyence BIOREVO All-in-One Fluorescence Microscope (BZ-9000, Keyence, Osaka, Japan) and transferred to Adobe Photoshop CS (Adobe, San Jose, CA, United States) to generate the figures. Contrast and brightness were adjusted with Adobe Photoshop CS. The lesion site and ventral horn at the fifth lumbar (L5) level were analyzed.

In the L5 ventral horn, *WFA*-positive and CS-A-positive PNN structures surrounding motoneurons positive for ChAT or NeuN were quantitatively analyzed in rats at 3 and 8 weeks after CST. We identified motoneurons for which 20% or more of the circumference was surrounded by CS-A-positive structures as positive for CS-A. We further divided ChAT-positive neurons into three groups: (1) those for which more than 80% of the circumference was surrounded by CS-A-positive structures, (2) those for which 20–80% of the circumference was surrounded by CS-A-positive structures, and (3) those for which less than 20% of the circumference was surrounded by CS-A-positive structures, as described in previous studies (Takiguchi et al., 2021, 2022). Similar criteria were applied to the *WFA*-positive structures. The number of motoneurons in each group was counted by researchers blinded to the group conditions. The percentage of motoneurons surrounded by CS-A-positive or *WFA*-positive structures among all motoneurons was then calculated for each of the three groups. This process was repeated for all rats. Statistical analysis was performed using a two-tailed unpaired *t*-test. A *p*-value of less than 0.05 was considered significant.

### Behavioral Analysis

The hindlimb locomotor functions of CST rats (*n* = 34) were assessed immediately before perfusion at 2, 3, and 8 weeks after CST. The Basso, Beattie, and Bresnahan (BBB) locomotor scale method (Basso et al., 1995) was calculated for each rat. The BBB scale (0–21) evaluates the recovery of hindlimb motor function after SCI by scoring motor function in terms of joint movement, hindlimb movement, stepping, and coordination between forelimbs and hindlimbs. Statistical analysis was performed using Student’s *t*-test. A *p*-value of less than 0.01 was considered significant.

## Results

### Motor Function of the Hindlimbs

**FIGURE 1:**
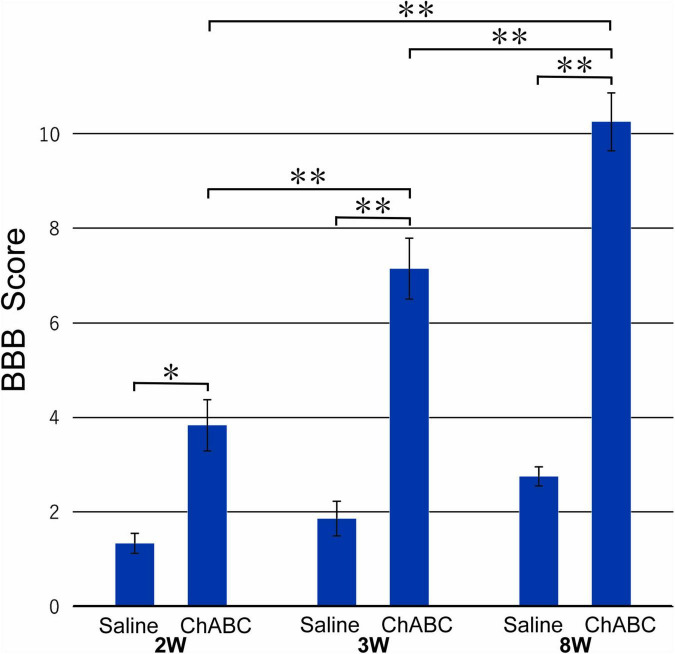
The Basso, Beattie, and Bresnahan (BBB) score of rats with ChABC or saline administration at 2, 3, and 8 weeks after complete spinal cord transection (CST). Significant differences are indicated by ^**^p < 0.01 or *p < 0.05.

The mean BBB score of the CST-ChABC rats was 3.83 ± 0.54 at 2 weeks after CST, 7.14 ± 0.64 at 3 weeks after CST, and 10.25 ± 0.61 at 8 weeks after CST. The mean BBB score of the CST-saline rats was 1.33 ± 0.21 at 2 weeks after CST, 1.86 ± 0.37 at 3 weeks after CST, and 2.75 ± 0.20 at 8 weeks after CST (Figure 1). The BBB scores of the CST-ChABC rats were significantly higher than those of CST-saline rats at 2, 3, and 8 weeks after CST, indicating better recovery.

### Axonal Regeneration of 5-Hydroxytryptamine Fibers Through the Lesion Site

#### Two and 3 Weeks After Complete Spinal Cord Transection

In the CST-ChABC rats at 2 weeks after CST, few 5-HT nerve fibers entered the lesion site. No 5-HT nerve fibers were observed in the nervous tissue caudal to the lesion site. At 2 weeks after CST, no 5-HT fibers entered the lesion site in the CST-saline rats.

**FIGURE 2:**
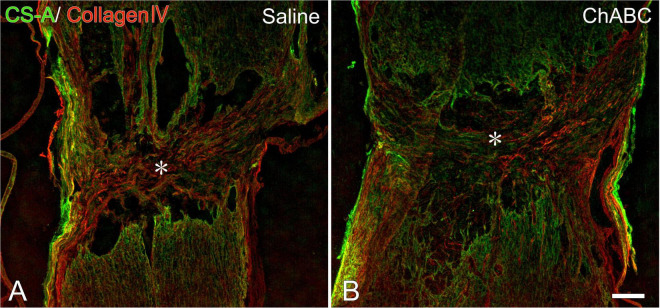
Double-labeled images of immunohistochemistry for collagen IV (red), chondroitin sulfate (CS)-A (green) at the lesion site of rats with saline **(A)**, and chondroitinase ABC (ChABC) **(B)** administration at 3 weeks after CST. Asterisks indicate the center of the lesion. Scale bars = 200 μm.

**FIGURE 3:**
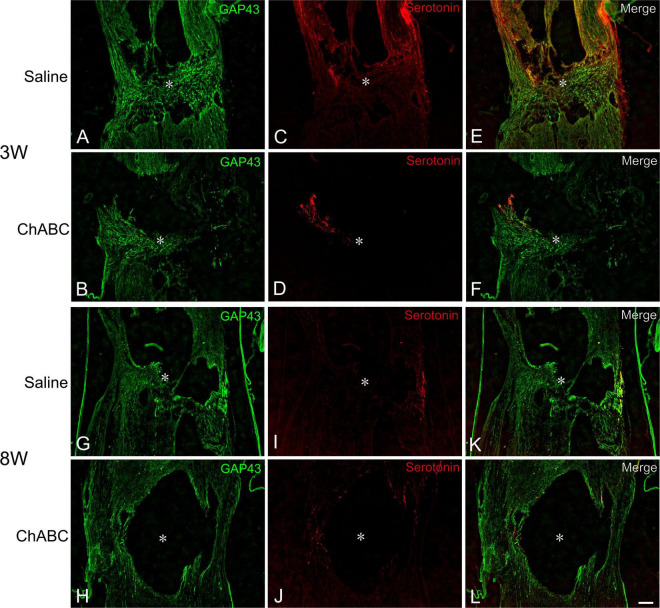
Double-labeled images of immunohistochemistry for 5-hydroxytryptamine (5-HT) (red), and GAP-43 (green) in the lesion site of rats with saline **(A,C,E,G,I,K)**, and ChABC **(B,D,F,H,J,L)** administration at 3 **(A–F)**, and 8 weeks **(G–L)** after CST. Images of GAP-43 **(A,B,G,H)**, 5-HT **(C,D,I,J)**, and merged images **(E,F,K,L)** are shown. Arrows indicate nerve fibers doubly positive for 5-HT and GAP-43. Asterisks indicate the center of the lesion site. Scale bars = 100 μm.

At 3 weeks after CST, scar tissue formed in both the CST-ChABC and CST-saline rats. The scar comprised fibrous tissue containing collagen IV-positive structures at the lesion level, and glial tissue containing CS-A-positive structures surrounded it from the rostral and caudal sides (Figure 2). In the CST-ChABC rats, many 5-HT nerve fibers entered the lesion site. Some 5-HT nerve fibers were also positive for GAP-43. Few 5-HT nerve fibers were observed in the nervous tissue caudal to the lesion site (Figures 3B,D,F). In the CST-saline rats, some 5-HT fibers entered the lesion site. They were also positive for GAP-43 (Figures 3A,C,E).

No 5-HT neurons were observed in the spinal cord, including the lesion site, in the CST rats at either 2 or 3 weeks after CST.

#### At 8 Weeks After Complete Spinal Cord Transection

In the CST-ChABC rats, many 5-HT nerve fibers passed through the fibrous tissue at the lesion site. These fibers crossed the caudal border of the lesion site and penetrated deep into the nervous tissue caudal to the lesion site. Some 5-HT fibers were observed in the meninges at the level of the lesion. Furthermore, 5-HT nerve fibers in the CST-ChABC rats were negative for GAP-43 (Figures 3H,J,L). In the CST-saline rats, many 5-HT nerve fibers entered deep into the lesion site. These fibers terminated near the caudal border of the lesion site but did not enter the nervous tissue caudal to the lesion site. Some 5-HT nerve fibers in the CST-saline rats were positive for GAP-43 (Figures 3G,I,K).

At 8 weeks after CST, no 5-HT neurons were observed in the spinal cord, including the lesion site.

#### Perineuronal Nets Around Motoneurons in the Fifth Lumbar Segment Ventral Horn

##### Chondroitin Sulfate-A-Positive Perineuronal Net Structures

**FIGURE 4:**
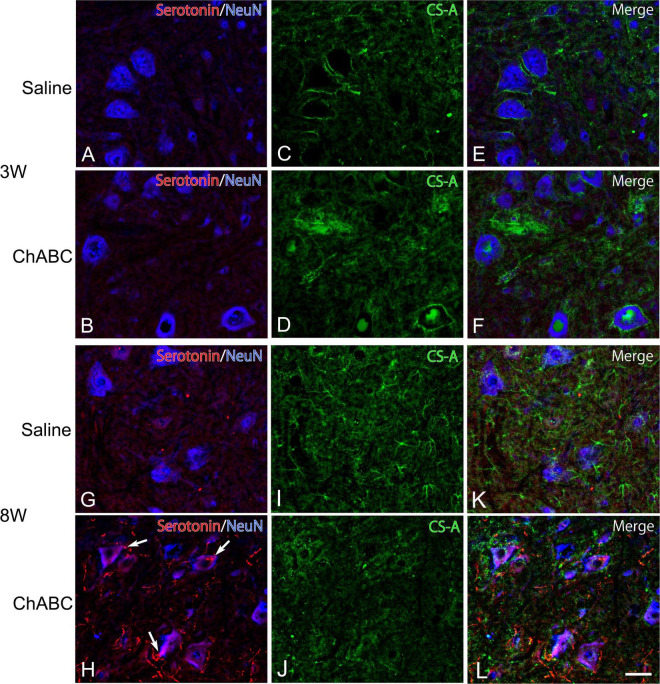
Images of immunohistochemistry for CS-A (green), Neu N (converted to blue), and 5-HT (red) in the L5 ventral horn of rats with saline **(A,C,E,G,I,K)**, and ChABC **(B,D,F,H,J,L)** administration at 3 **(A–F)**, and 8 **(G–L)** weeks after CST. Double-labeled images of 5-HT and NeuN **(A,B,G,H)**, CS-A immunohistochemistry images **(C,D,I,J)**, and merged images **(E,F,K,L)** are shown. Arrows in **(H)** indicate 5-HT nerve terminals on NeuN-positive motoneurons. Many 5-HT varicosities were also observed in **(H,L)**. Scale bars = 50 μm.

**FIGURE 5:**
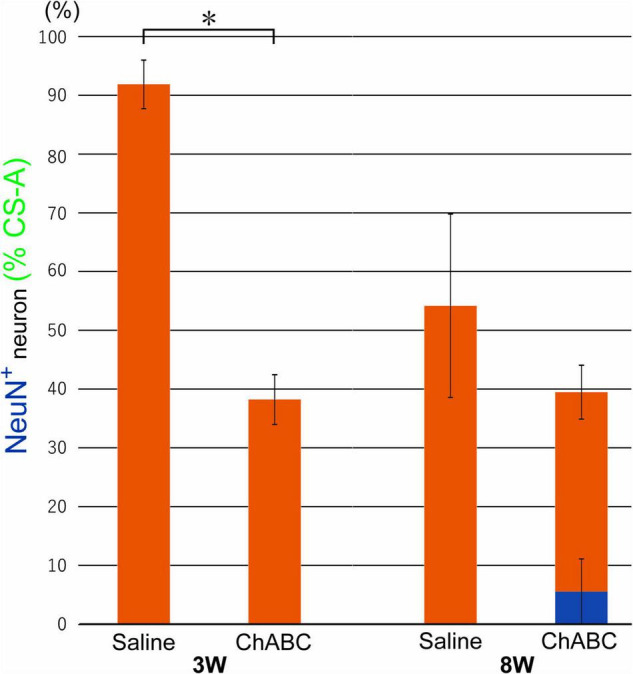
Percentages of motoneurons whose cell bodies were covered with CS-A-positive perineuronal net (PNN) structures among all motoneurons at 3 and 8 weeks after CST in rats with ChABC or saline administration. The blue bar indicates the percentage of motoneurons with > 80% CS-A-positive structures and the red bars indicate the percentage of motoneurons with > 20% CS-A-positive structures. Data are expressed as the mean ± SEM. Significant differences are indicated by *(*p* < 0.05).

Chondroitin sulfate-A-positive structures were observed around motoneurons in the L5 ventral horn at 2, 3, and 8 weeks after CST (Figure 4). The percentages of motoneurons covered with CS-A-positive structures in the CST-ChABC rats were 38.23 ± 4.24% at 3 weeks after CST, and 39.48 ± 9.53% at 8 weeks after CST. The percentages of motoneurons covered with CS-A-positive structures in the CST-saline rats were 91.85 ± 4.12% at 3 weeks after CST and 54.18 ± 15.18% at 8 weeks after CST (Figure 5).

##### Wisteria Floribunda Agglutinin-Positive Perineuronal Net Structures

**FIGURE 6:**
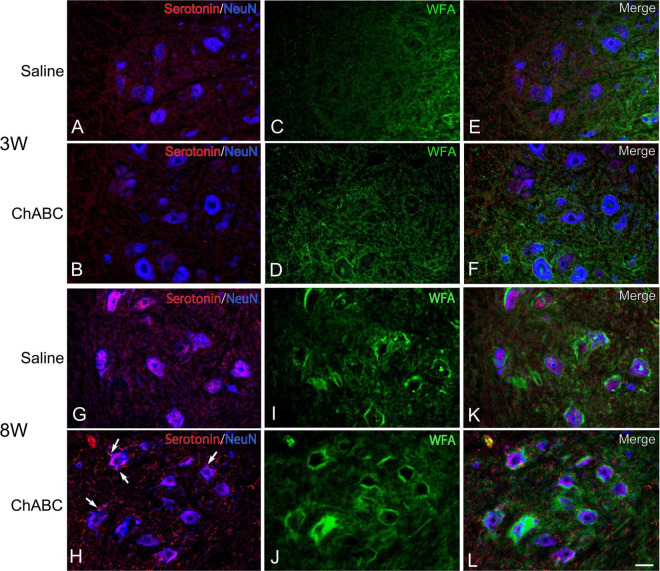
Images of *Wisteria Floribunda agglutinin (WFA)* histochemistry (green), and immunohistochemistry for NeuN (converted to blue), and 5-HT (red) in the L5 ventral horn of rats with saline **(A,C,E,G,I,K)**, and ChABC **(B,D,F,H,J,L)** administration after 3 **(A–F)** and 8 **(G–L)** weeks after CST. Double-labeled images of 5-HT and NeuN **(A,B,G,H)**, *WFA* histochemistry images **(C,D,I,J)**, and merged images **(E,F,K,L)** are shown. Arrows in **(H)** indicate 5-HT nerve terminals on NeuN-positive motoneurons. Many 5-HT varicosities were also observed in **(H,L)**. Scale bars = 50 μm.

**FIGURE 7:**
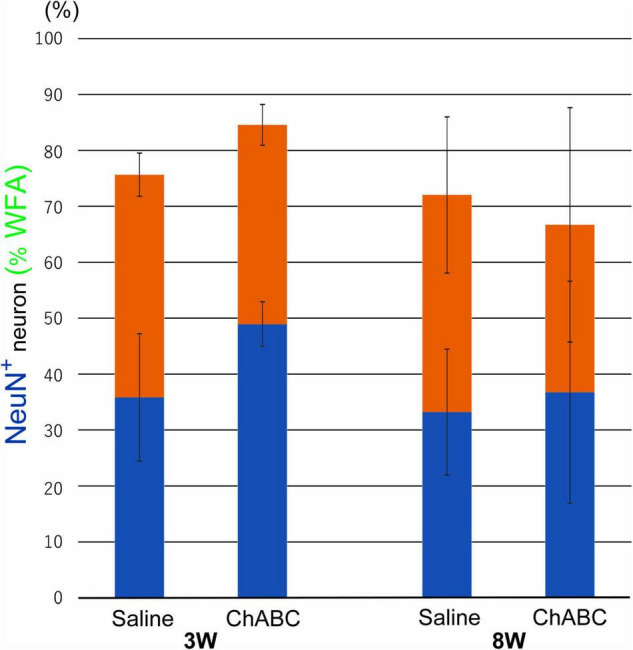
Percentages of motoneurons whose cell bodies were covered with *WFA*-positive PNN structures among all motoneurons at 3 and 8 weeks after CST in rats with ChABC or saline administration. Blue bars indicate the percentage of motoneurons with > 80% *WFA*-positive structures, and red bars indicate the percentage of motoneurons with > 20% *WFA*-positive structures. Data are expressed as the mean ± SEM.

*Wisteria Floribunda agglutinin*-positive structures were observed around motoneurons in the L5 ventral horn at 2, 3, and 8 weeks after CST (Figure 6). The percentages of motoneurons covered with *WFA*-positive structures in the CST-ChABC rats were 84.58 ± 3.65% at 3 weeks after CST and 66.71 ± 20.94% at 8 weeks after CST. The percentages of motoneurons covered with *WFA*-positive structures in the CST-saline rats were 75.66 ± 3.84% at 3 weeks after CST and 72.04 ± 13.96% at 8 weeks after CST (Figure 7).

##### 5-Hydroxytryptamine Nerve Terminals in the Fifth Lumbar Segment Ventral Horn at 8 Weeks After Complete Spinal Cord Transection

**FIGURE 8:**
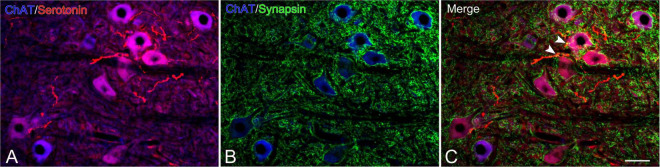
Images of immunohistochemistry for synapsin I (green), ChAT (converted to blue), and 5-HT (red) in the L5 ventral horn of rats with ChABC administration at 8 weeks after CST. Double-labeled image of ChAT and 5-HT **(A)**, ChAT and synapsin I **(B)**, and merged images **(C)**. Arrowheads in **(C)** indicate nerve terminals doubly positive for 5-HT and synapsin I on motoneurons. Scale bars = 50 μm.

In the CST-ChABC rats, many 5-HT nerve fibers were observed in the L5 ventral horn. The termination of 5-HT nerve fibers on motoneurons positive for ChAT or NeuN was frequently observed (Figures 4, 6). Some of these 5-HT terminals were colocalized with synapsin I (Figure 8). 5-HT nerve terminals were in contact with motoneurons covered with CS-A-positive structures as well as those that were not covered with CS-A-positive structures (Figure 4). Similarly, 5-HT nerve terminals contacted motoneurons covered with *WFA*-positive structures and motoneurons not covered with WFA-positive structures (Figure 6). In the CST-saline rats, no 5-HT nerve fibers terminals in contact with motoneurons in the L5 ventral horn were observed (Figures 4, 6).

## Discussion

The degradation of CS-GAGs by the administration of ChABC efficiently promotes axonal regeneration and spinal neuronal plasticity and is applied as a therapeutic strategy for SCI (Bradbury et al., 2002; Yick et al., 2004; Caggiano et al., 2005; Barritt et al., 2006; Huang et al., 2006; García-Alías et al., 2008; Tester and Howland, 2008; Bradbury and Carter, 2011; Starkey et al., 2012; Muir et al., 2019). In the present study, ChABC administration at the lesion site immediately after CST in rats promoted the passage of descending 5-HT axons through the fibrous scar at the lesion site and termination of the lumbar motoneurons.

The descending 5-HT projections to the spinal cord originate in neurons in the nucleus raphe magnus (B3), raphe obscurus (B2), and raphe pallidus (B1) in the medulla oblongata. Among them, the raphe obscurus projects 5-HT axons to the ventral horn. In addition, 5-HT neurons have enhanced regenerative abilities that activate axonal sprouting and regeneration after SCI. In incomplete SCI models, such as contusion or hemisection, many sprouted axons from spared 5-HT axons and regenerated axons from injured 5-HT axons pass the lesion site to project to the level caudal to the lesion (Perrin and Noristani, 2019). In the CST model, on the other hand, 5-HT axons do not regenerate beyond the lesion site, and the axons caudal to the lesion site are lost permanently (Perrin and Noristani, 2019). This was confirmed by the present results in control CST rats with saline administration. Furthermore, no 5-HT neurons were observed at the lesion site or in the spinal cord caudal to the lesion site in either the CST-saline rats or CST-ChABC rats, suggesting that the appearance of 5-HT neurons induced by injury, as reported in some vertebrates, is impossible (Takeda et al., 2008; Kuscha et al., 2012; Fabbiani et al., 2018). The present findings in rats with ChABC administration, therefore, suggest that axons from brainstem 5-HT neurons have the regenerative ability to pass through the lesion site under the influence of ChABC. The mechanisms underlying the successful regeneration of 5-HT axons beyond the lesion site are unclear. A previous study demonstrated that 5-HT axons are more capable of surviving and sprouting than cortical neurons under the inhibitory environment of CSPGs, suggesting that 5-HT axons have higher endogenous growth activity than other axonal types (Hawthorne et al., 2011). Furthermore, 5-HT axons express high levels of proteases that degrade CSPGs (Tran et al., 2018). The degradation of CS-GAGs by ChABC administration might support the endogenous activity of 5-HT axons to overcome the inhibitory environment of CSPGs at the lesion site. The present findings also showed that 5-HT axons terminated on motoneurons in the L5 in rats with ChABC administration. Many 5-HT terminals co-localized with synapsin I, suggesting that they made synaptic contacts with motoneurons. Further quantitative studies of synapse formation, however, are needed to elucidate the details of reinnervation of 5-HT axons.

The present behavioral analysis showed that hindlimb locomotor activities of CST-ChABC rats at 2, 3, and 8 weeks after CST rats were significantly improved compared with those of CST-saline rats. At 8 weeks after CST, many 5-HT axon terminals were observed on motoneurons in the L5 ventral horn of rats following ChABC administration, suggesting that regenerated 5-HT projections to motoneurons may contribute to the recovery of hindlimb locomotor activities. In the L5 ventral horn, however, many 5-HT varicosities were not in contact with neurons. Previous studies have shown that 5-HT neurotransmission predominantly occurs *via* a volumetric transmission that involves 5-HT diffusion across the extracellular space (Séguéla et al., 1989; Oleskevich et al., 1991; Miner et al., 2000). Therefore, even 5-HT terminals that did not make synaptic contact with ventral horn neurons might have contributed to the recovery of locomotor function of the CST-ChABC rats at 8 weeks after CST. Our observations in the CST-ChABC rats showed that 5-HT terminals in the L5 ventral horn were few at 2 and 3 weeks after CST, although the hindlimb locomotor activity was recovered. Diffusion of 5-HT released from regenerated axons at levels rostral to the L5 might have contributed to the recovery of locomotor activities. Alternatively, it is possible that the regeneration of descending tracts and sprouting within the spinal cord by neurons other than 5-HT neurons was involved in the recovery of locomotor function in the CST-ChABC rats. Retrograde tracing in the CST-ChABC rats could show populations of neurons projecting to the L5 ventral horn. It is also possible that the axonal sprouting of primary afferents contributed to the recovery of locomotor function. The primary afferent projections to the ventral horn and intermediate zone in L5 are strengthened after thoracic CST in neonatal rats (Takiguchi et al., 2015). Further experiments are needed to evaluate these possibilities.

Perineuronal nets are ECM structures that surround neuronal cell bodies and proximal dendrites in the central nervous system. The main component of PNN is CSPG, which contains CS-GAG, a major inhibitory factor that contributes to neural plasticity and development (Caterson, 2012; Soleman et al., 2013; Dyck and Karimi-Abdolrezaee, 2015). The present results showed that the percentage of motoneurons with CS-A-positive structures in the CST-ChABC rats was significantly smaller than that in the CST-saline rats at 3 weeks after CST. This finding suggests that ChABC applied to the lesion site successfully degraded CS-GAGs around motoneurons in the L5 ventral horn. ChABC administration, therefore, might facilitate the reinnervation of 5-HT axons by promoting the plasticity of motoneurons. On the other hand, the percentage of motoneurons with *WFA*-positive structures in the CST-ChABC rats did not significantly differ from that in the CST-saline rats at 3 weeks after CST, suggesting that ChABC applied to the lesion site had little effect on PNNs around motoneurons in the L5 ventral horn. Therefore, regenerated 5-HT axons might make synaptic contacts with motoneurons surrounded by PNNs when CS-GAGs are degraded to some extent by ChABC. At 8 weeks after CST, 5-HT terminals were observed on both motoneurons with and without *WFA*-positive structures in rats with ChABC administration.

In this study, 50 μl of ChABC solution (1 U/ml) was soaked in a gelatin sponge and applied to the lesion site immediately after the CST. The methods of ChABC administration in previous studies vary in terms of the sites of administration, timing, dose, and duration (Muir et al., 2019). Intraparenchymal injection of high, but not low, doses of ChABC 2 weeks after lesioning promotes axonal regeneration across the lesion site, resulting in the recovery of locomotor activities in rats with thoracic CST (Cheng et al., 2015). These results suggest that a high dose of ChABC delivered during the subacute phase after CST is most effective for axonal regeneration and functional recovery (Muir et al., 2019). On the other hand, the present results revealed that slow release of a low dose of ChABC also promotes axonal regeneration beyond the lesion site after CST, and results in a more pronounced functional recovery compared with high-dose injection, which leads to only a modest functional recovery (an average BBB score of 3) (Cheng et al., 2015). CSPGs and their components are dramatically increased at the lesion site within days after hemisection or contusion of the spinal cord in adult rats (Lemons et al., 1999; Jones et al., 2003; Tang et al., 2003). Considering that the intraparenchymal administration of a low dose of ChABC 2 weeks after CST did not promote axonal regeneration (Cheng et al., 2015), our findings suggest that the application of ChABC immediately after CST effectively degrades CSPGs and promotes axonal regeneration.

## Data Availability Statement

The original contributions presented in this study are included in the article, further inquiries can be directed to the corresponding author/s.

## Ethics Statement

The animal study was reviewed and approved by the Institutional Animal Care and Use Committee of the Animal Research Center of Yokohama City University (Approval No. F-A-21-001).

## Author Contributions

MT: methodology, resources, investigation, validation, formal analysis, visualization, and writing—original draft. KM and KY: investigation, formal analysis, and visualization. KF: conceptualization, data curation, writing—original draft, and funding acquisition. All authors contributed to the article and approved the submitted version.

## Conflict of Interest

The authors declare that the research was conducted in the absence of any commercial or financial relationships that could be construed as a potential conflict of interest.

## Publisher’s Note

All claims expressed in this article are solely those of the authors and do not necessarily represent those of their affiliated organizations, or those of the publisher, the editors and the reviewers. Any product that may be evaluated in this article, or claim that may be made by its manufacturer, is not guaranteed or endorsed by the publisher.
